# 3-Methyl-4-nitrophenol Exposure Deteriorates Oocyte Maturation by Inducing Spindle Instability and Mitochondrial Dysfunction

**DOI:** 10.3390/ijms25073572

**Published:** 2024-03-22

**Authors:** Fan Chen, An-Feng Luo, Ming-Guo Li, Li-Xiang Zheng, Hao Gu, Chang-Fan Zhou, Wei Zeng, Adrian Molenaar, Hong-Yan Ren, Yan-Zhen Bi

**Affiliations:** 1 Key Laboratory of Animal Embryo Engineering and Molecular Breeding of Hubei Province, Institute of Animal Sciences and Veterinary Medicine, Hubei Academy of Agricultural Sciences, Wuhan 430070, China; fanchen@hbaas.com (F.C.); laf47630365@163.com (A.-F.L.); 16699230598@163.com (M.-G.L.); zhenglx0712@163.com (L.-X.Z.); guhao@hbaas.com (H.G.); zhouchangfan@webmail.hzau.edu.cn (C.-F.Z.); adrian.molenaar@agresearch.co.nz (A.M.); 2 Rumen Microbiology and Animal Nutrition and Physiology AgResearch, Grasslands Campus, Fitzherbert Research Centre, Palmerston North 4410, New Zealand

**Keywords:** oocyte maturation, PNMC, spindle stability, Fignl1, mitochondria

## Abstract

3-methyl-4-nitrophenol (PNMC), a well-known constituent of diesel exhaust particles and degradation products of insecticide fenitrothion, is a widely distributed environmental contaminant. PNMC is toxic to the female reproductive system; however, how it affects meiosis progression in oocytes is unknown. In this study, in vitro maturation of mouse oocytes was applied to investigate the deleterious effects of PNMC. We found that exposure to PNMC significantly compromised oocyte maturation. PNMC disturbed the spindle stability; specifically, it decreased the spindle density and increased the spindle length. The weakened spindle pole location of microtubule-severing enzyme Fignl1 may result in a defective spindle apparatus in PNMC-exposed oocytes. PNMC exposure induced significant mitochondrial dysfunction, including mitochondria distribution, ATP production, mitochondrial membrane potential, and ROS accumulation. The mRNA levels of the mitochondria-related genes were also significantly impaired. Finally, the above-mentioned alterations triggered early apoptosis in the oocytes. In conclusion, PNMC exposure affected oocyte maturation and quality through the regulation of spindle stability and mitochondrial function.

## 1. Introduction

Owing to multiple factors, including environmental pollution, mental stress, and delayed child-bearing age, female infertility has emerged as a global problem, affecting nearly 15% of couples worldwide [1]. The quality of the oocyte is a decisive factor for female fertility, as it subsequently affects fertilization, embryonic development, and implantation [2]. Unlike somatic cells, mammalian oocytes are dormant at the meiotic diplotene stage of prophase before birth, lasting for months in animals and decades in humans. Until each menstrual or estrus cycle, stimulated by follicle-stimulating and luteinizing hormones, oocytes resume meiosis and successively undergo landmark events, including germinal vesicle breakdown (GVBD), spindle organization, homologous chromosomes alignment and segregation [3]. Oocytes are more sensitive to external toxic insults owing to these long periods of meiotic dormancy [4].

3-methyl-4-nitrophenol (4-nitro-m-cresol, PNMC), a well-known component of diesel exhaust particles, causes serious air pollution [5]. PNMC is also the main degradation product of fenitrothion, a commonly used pesticide in agriculture [6]. Moreover, PNMC is widely used in the production of fungicides, drugs, dyes, and rubber materials [7]. Owing to its widespread use, PNMC has emerged as a common contaminant worldwide and is ubiquitous in the air, food, soil, and water. Humans are prone to PNMC exposure through inhalation, orally, or through the skin [8]. As its degradation is difficult, PNMC persistently remains in the environment. The United States Environmental Protection Agency has listed PNMC under “Priority control pollutants” [9].

PNMC exerts adverse effects on human and animal health. It induces the development of airway diseases, breast adenocarcinoma, and immune disease [10,11,12,13]. As a nitrophenol derivative, PNMC is an endocrine-disrupting chemical and shows estrogenic and antiandrogenic activity [14,15,16]. PNMC exposure causes serious reproductive damage in animals by disrupting the endocrine function and increasing the number of apoptotic testicular cells in male quails, rats, chickens, and mice [17,18]. Recently published data suggest that PNMC is a negative modulator of steroidogenesis in chicken preovulatory follicles [19,20]. PNMC treatment impairs oocyte meiotic progression and follicle development in murine models [21].

PNMC exposure can hinder meiotic maturation in oocytes; however, subsequent effects and the underlying mechanism by which PNMC affects the oocyte quality remain unknown [21]. This study attempted to elaborate on the mechanism underlying the effects of PNMC on the maturation of murine oocytes. Taking advantage of an oocyte in vitro culture system, we showed that a low-dose PNMC treatment (50 nM) seriously perturbed oocyte maturation and quality. Our results directly validated that PNMC exposure broke spindle stability and mitochondrial function, finally triggered early apoptosis in oocytes.

## 2. Results

### 2.1. PNMC Exposure Compromises the Meiotic Maturation

**Figure 1:**
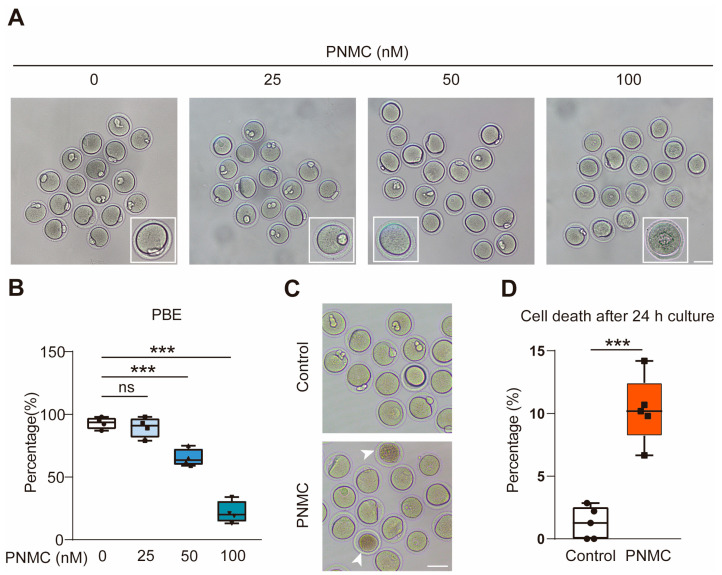
PNMC exposure disturbs the meiotic maturation of oocytes. (**A**) Fully grown GV oocytes were exposed to PNMC at the indicated concentrations (0, 25, 50, and 100 nM) for 14 h. Control and 25 nM PNMC-treated groups normally attained meiotic maturation; however, most oocytes failed to accomplish PBE after treatment with 50 and 100 nM PNMC. Scale Bar = 100 µm. (**B**) The percentages of PBE in control (*n* = 206) and PNMC-exposed groups (25 nM, *n* = 255; 50 nM, *n* = 281; 100 nM, *n* = 196) are shown. (**C**) The oocytes were cultured for 24 h to investigate the effects of PNMC exposure on oocyte mortality. Cell death was prominent in the 50 nM PNMC-exposed oocytes, unlike in the control group. Scale Bar = 100 µm. (**D**) The proportion of cell death was analyzed in control (*n* = 195) and 50 nM PNMC-exposed (*n* = 188) oocytes. ns (not significant) means *p* ≥ 0.05; *** *p* < 0.001.

The first polar body extrusion (PBE) at meiosis I marks the meiotic maturation of oocytes. Therefore, germinal vesicle (GV) oocytes were grown in a medium supplemented with 0, 25, 50, and 100 nM of PNMC to observe PBE in vitro. As shown in Figure 1A,B, PNMC treatment significantly diminishes the PBE rate of oocytes in a dose-dependent manner. The result validated that the rate of PBE was not significantly affected in the 25 nM PNMC group (*p* > 0.05). Remarkably, a significant decrease was observed in the 50 and 100 nM PNMC groups (*p* < 0.001). After 24 h exposure to 50 nM PNMC, cell death increased significantly (*p* < 0.001; Figure 1C,D) compared with control oocytes. Subsequently, 50 nM PNMC was chosen to explore the toxic mechanisms in this study, as it could significantly inhibit PBE but allow a few oocytes to normally attain maturation.

### 2.2. PNMC Exposure Impairs Spindle Stability

**Figure 2:**
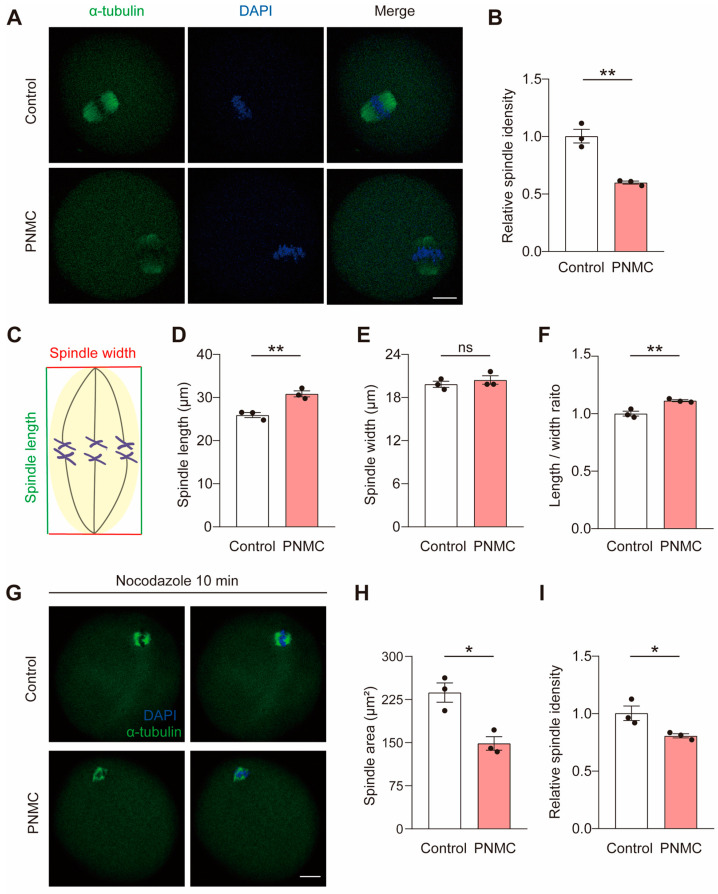
PNMC exposure causes spindle instability at the MI stage. (**A**) The representative images of spindle morphology and chromosome alignment in control and PNMC-exposed oocytes. After excluding the oocytes that did not accomplish GVBD at 2 h, the remaining control and PNMC-treated oocytes were immune-stained with α-tubulin after another 6 h culture (MI oocytes). α-tubulin, green; DNA, blue. Scale Bar = 20 µm. (**B**) The fluorescence intensity of spindle α-tubulin was quantified in control (*n* = 26) and PNMC-exposed (*n* = 29) oocytes. (**C**–**F**) Spindle length, width, and length/width ratio were quantified in control (*n* = 36) and PNMC-exposed (*n* = 38) groups. (**G**) Images delineating spindle morphology in control and PNMC-exposed MI oocytes after nocodazole treatment. α-tubulin, green; DNA, blue. Scale Bar = 20 µm. (**H**,**I**) The spindle fluorescence intensity and area were quantified in control (*n* = 28) and PNMC-exposed (*n* = 31) groups. * *p* < 0.05, ** *p* < 0.01.

We evaluated the spindle architecture and chromosome alignment at metaphase I (MI) oocytes to determine how PNMC exposure disrupted the meiotic progression. Interestingly, homologous chromosomes were well-aligned at the metaphase plate in the PNMC-exposed oocytes but the spindle apparatus showed moderate defects, confirming a diminished spindle microtubule integrity in the vicinity of chromosomes (Figure 2A,B). The lengths of the spindles increased significantly in PNMC-exposed groups (30.85 ± 0.99 µm vs. 25.93 ± 0.82 µm; *p* < 0.01; Figure 2C,D); however, the spindle width was uniformly consistent in control oocytes (20.43 ± 0.83 µm vs. 19.84 ± 0.60 µm; *p* > 0.05; Figure 2E). Consistently, the ratio of spindle length/width increased significantly after PNMC exposure (Figure 2F).

Given the spindle defects after PNMC exposure, we used nocodazole, a reversible microtubule depolymerized agent, to confirm the effects of PNMC on spindle stability. After 10 min of nocodazole depolymerization, the area of spindle microtubules in MI-stage oocytes in the PNMC-exposed group was significantly smaller than in controls (237.20 ± 23.75 µm^2^ vs. 148.71 ± 16.65 µm^2^; *p* < 0.05; Figure 2G,H), along with reduced fluorescence intensity (Figure 2I). Therefore, 50 nM PNMC treatment disrupted spindle stability but not the chromosomal alignment.

### 2.3. PNMC Exposure Diminishes the Localization Signal of Fignl1 at the Spindle Poles

**Figure 3:**
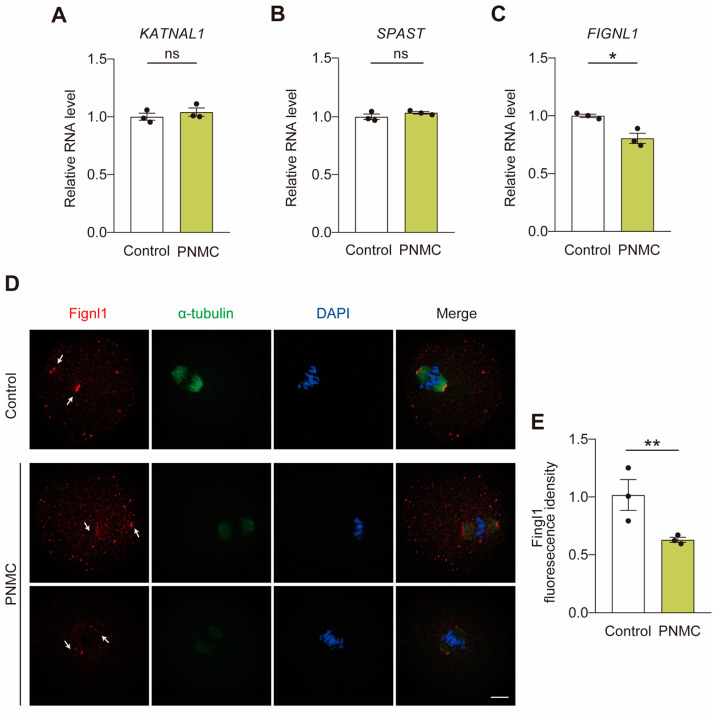
PNMC exposure disturbs spindle localization of Fignl1 in oocytes. (**A**–**C**) The mRNA levels of *KATNAL1*, *FIGNL1*, and *SPAST* were confirmed by qRT-PCR. The mRNA expressions of *SPAST* and *KATNAL1* were unaffected at the MI stage between control and PNMC-exposed groups; however, the level of *FIGNL1* was significantly inhibited after PNMC exposure. (**D**) Images illustrating the localization pattern of Fignl1 in control and PNMC-exposed MI oocytes. α-tubulin, green; Fignl1, red; DNA, blue. Scale Bar = 20 µm. (**E**) Quantitative analysis of the fluorescence intensity of Fignl1 in control (*n* = 28) and PNMC-exposed (*n* = 30) groups. * *p* < 0.05, ** *p* < 0.01.

Ablation of microtubule-severing proteins (MTSPs) induces defects in the spindle apparatus in oocytes, consistent with the aberrant phenotype observed after PNMC exposure. Therefore, the effect of PNMC treatment on the expression of MTSP marker genes (*SPAST*, *KATNAL1*, and *FIGNL1*) was evaluated by qRT-PCR. The mRNA levels of *SPAST* and *KATNAL1* exhibited no significant difference in the MI stage between control and PNMC-exposed groups (Figure 3A,B); however, the mRNA levels of *FIGNL1* were significantly lower after PNMC exposure (Figure 3C).

We investigated if the normal localization of the Fignl1 to the spindle pole was affected at the MI stage after PNMC exposure. As shown in Figure 3D, strong fluorescence signals of Fignl1 are found at the spindle poles in control oocytes. In contrast, the sharply weak signal of Fignl1 at spindle poles is found in PNMC-treated oocytes. Quantitative analysis confirmed that the fluorescent intensity of Fignl1 decreased significantly in the PNMC-exposed oocytes compared to the control oocytes (Figure 3E). Taken together, PNMC exposure damaged localization pattern of Fignl1 at spindle poles, possibly resulting in the defective assembly of the spindle apparatus after PNMC exposure.

### 2.4. PNMC Exposure Disrupts Mitochondrial Function

**Figure 4:**
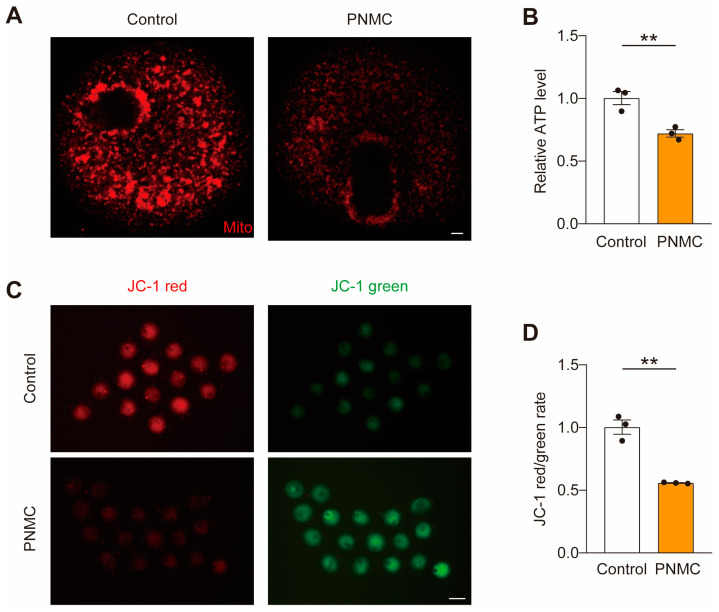
PNMC exposure disrupts the mitochondrial function in oocytes. (**A**) Control and PNMC-treated MI oocytes were labeled with MitoTracker Red to visualize mitochondrial distribution. Scale Bar = 5 µm. (**B**) ATP levels were determined in the control (*n* = 43) and PNMC-exposed (*n* = 39) groups. (**C**) MMP in the control and PNMC-exposed MI oocytes by JC-1 staining. The green signal represents inactive mitochondria and the red signal represents active mitochondria in oocytes. Scale Bar = 100 µm. (**D**) MMP was quantified in control (*n* = 30) and PNMC-exposed (*n* = 28) groups. ** *p* < 0.01.

Given the crosstalk between spindle and mitochondria, we examined if the mitochondrial function was impaired after PNMC exposure. Mitochondrial accumulation at both cytoplasm and spindle peripheries decreased in most PNMC-exposed oocytes (Figure 4A), indicating that PNMC disrupted the mitochondrial distribution during oocyte maturation. Next, we examined ATP production. ATP levels in the PNMC-exposed oocytes were significantly lower than those in the control oocytes (Figure 4B). Consistently, the mitochondrial membrane potential (MMP), which drives ATP synthesis, was significantly reduced in PNMC-treated oocytes (Figure 4C,D). Therefore, PNMC exposure impaired mitochondrial function, including mitochondria distribution, ATP production, and MMP.

### 2.5. PNMC Exposure Elevated the ROS Level

**Figure 5:**
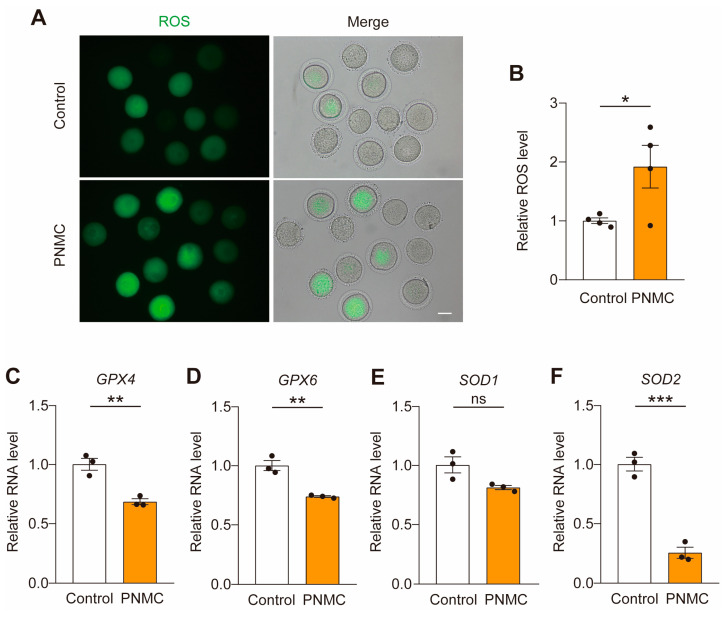
PNMC treatment induced the increased ROS levels in mouse oocytes. (**A**) ROS levels were assessed by DCFH-DA (green) staining. Representative images of ROS production in the control and PNMC-exposed MI oocytes. Scale Bar = 50 µm. (**B**) ROS levels were quantified in the control (*n* = 46) and PNMC-exposed (*n* = 38) groups. (**C**–**F**) The mRNA levels of antioxidant genes were evaluated by qRT-PCR. * *p* < 0.05, ** *p* < 0.01 and *** *p* < 0.001.

Mitochondria are the center of oxidative metabolism and the principal site of reactive oxygen species (ROS) production. ROS levels increased if mitochondrial dynamics and function were disrupted. The ROS levels increased in the PNMC-exposed groups (Figure 5A,B). Consistent with the elevated ROS levels, decreased expression of antioxidant genes (*GPX4*, *GPX6* and *SOD2*) was confirmed in the PNMC-exposed oocytes (Figure 5C–F). So, PNMC exposure leads to the accumulation of ROS.

### 2.6. PNMC Exposure Decreases the Expression of Mitochondria-Related Genes

**Figure 6:**
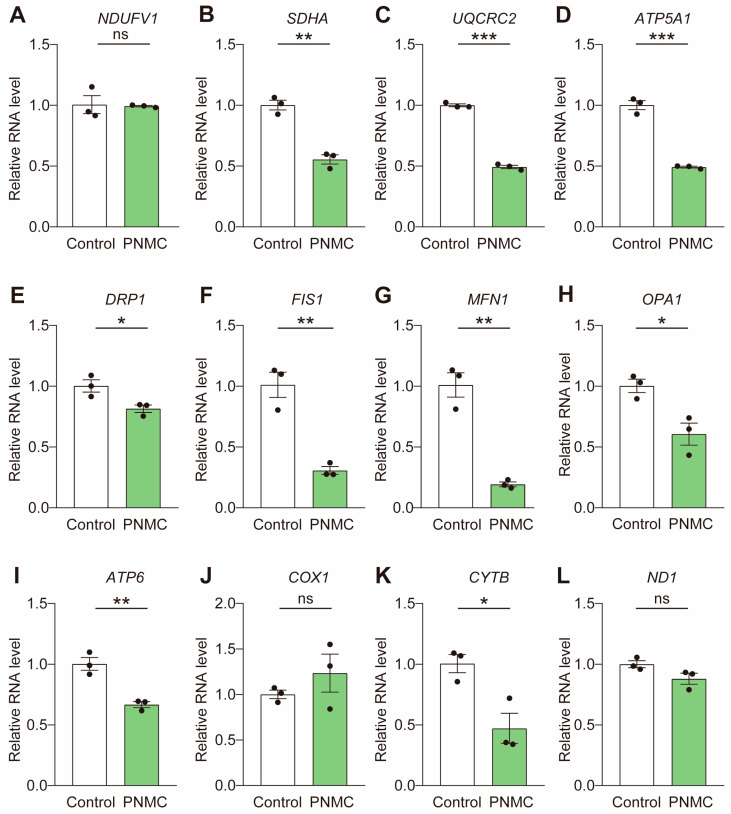
PNMC exposure blocks the expression of mitochondrial-related genes. (**A**–**D**) The relative mRNA levels of mitochondrial respiratory complexes, including *SDHA*, *UQCRC2*, and *ATP5A1*, were significantly decreased in PNMC-exposed oocytes. (**E**–**H**) The relative mRNA levels of genes related to mitochondrial dynamics, *DRP1*, *FIS1*, *MFN1*, and *OPA1*, were sharply reduced in PNMC-exposed oocytes. (**I**–**L**) The mtDNA, *ATP6* and *CYTB*, were significantly down-regulated after PNMC exposure. * *p* < 0.05, ** *p* < 0.01 and *** *p* < 0.001.

Given mitochondrial dysfunction upon exposure to PNMC, we examined the expression levels of mitochondria-related genes. First, we determined the mRNA levels of the mitochondrial respiratory complexes. As shown in Figure 6A–D, the transcriptional profiles of *SDHA* (Complex II), *UQCRC2* (Complex III), and *ATP5A1* (Complex V) were significantly decreased, explaining the reduced ATP levels in PNMC-exposed oocytes. We assessed the transcriptional profiles of mitochondria fission genes, *FIS1* and *DRP1*, and fusion genes, *MFN1* and *OPA1*. The mRNA levels of these genes were all significantly diminished (Figure 6E–H). The aberrant mitochondrial dynamic (fission and fusion) may have been partially caused by the defective mitochondrial distribution in PNMC oocytes. PNMC exposure significantly downregulated the mRNA levels of mitochondrial DNA (mtDNA), *ATP6* and *CYTB* (Figure 6I–L). Taken together, PNMC may directly target the mitochondria.

### 2.7. PNMC Exposure Triggers Early Apoptosis

**Figure 7:**
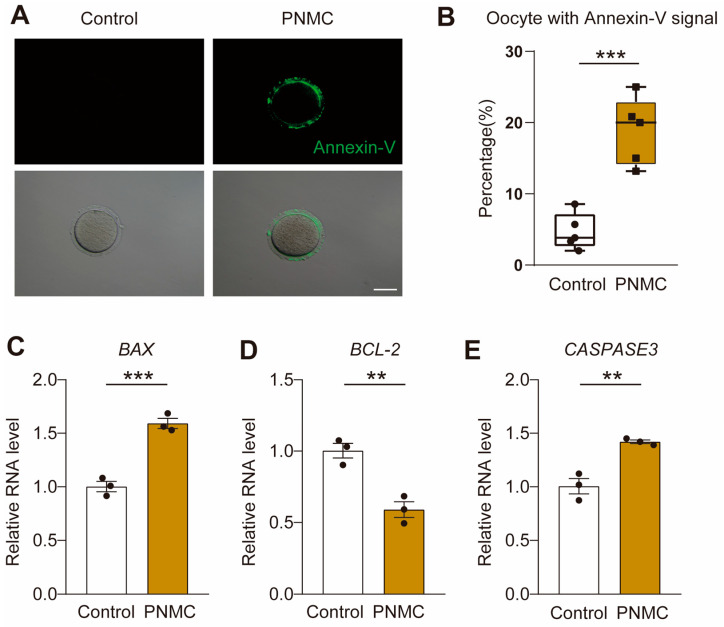
PNMC treatment triggers early apoptosis in oocytes. (**A**) Early apoptosis in the control and PNMC-exposed MI oocytes was evaluated by Annexin-V assay. Annexin-V, green; Scale Bar = 100 µm. (**B**) The proportion of the oocytes with Annexin-V positive signal was quantified in the control (*n* = 187) and PNMC-exposed (*n* = 194) oocytes. (**C**–**E**) qRT-PCR for the mRNA levels of *BAX*, *BCL-2*, and *CASPASE3* in the control and PNMC-exposed oocytes. ** *p* < 0.01 and *** *p* < 0.001.

Owing to the aberrant mitochondrial function following PNMC exposure, we speculated that the apoptotic level of oocytes may also be elevated. Therefore, we performed Annexin-V-FITC staining analysis to examine the apoptosis level. In the control groups, there were almost no apoptotic oocytes. However, the number of Annexin-V-positive oocytes increased significantly in the PNMC-exposed group (*p* < 0.05; Figure 7A,B). The mRNA levels of the apoptotic genes, *BAX* and *CASPASE3*, were significantly up-regulated. The mRNA level of the anti-apoptotic gene, *BCL-2*, was remarkably down-regulated in the PNMC-exposed oocytes (Figure 7C–E). We inferred that PNMC treatment triggered early apoptosis in mouse oocytes.

## 3. Discussion

In female reproduction, the quality of the oocyte is the key determinant of fertilization and embryo developmental competence; however, it is vulnerable to environmental factors. As an endocrine-disrupting chemical, PNMC is a global cause of concern owing to its adverse effects on human and animal health, especially reproductive toxicity [18,19]. PNMC exposure impairs the meiotic maturation of oocytes; however, the specific effect and underlying mechanism are not elusive. In this study, we sought to elaborate on the mechanisms underlying the effects of PNMC on the maturation of murine oocytes. Our findings suggest that PNMC exposure disturbs spindle organization and mitochondrial function, ultimately disrupting the first PBE and triggering early apoptosis in oocytes.

PNMC exposure caused spindle defects in oocytes, which were evident through decreased spindle density and increased spindle length. Nocodazole treatment confirmed that aberrant spindle architecture impaired spindle microtubule stability after PNMC exposure. The precise spindle microtubule organization is important for meiosis progression. It is regulated by various microtubule-associated proteins (MAPs) [22]. MTSPs are important MAPs and belong to the AAA-ATPases enzyme family, which uses the chemical energy of ATP-hydrolysis to sever microtubules [23,24]. MTSPs are important in spindle formation during mitosis and meiosis as they regulate the length, mass, and density of spindle microtubules, including Katanin, Spastin, and Fidgetin [25,26]. As spindle defects after PNMC exposure are consistent with MTSP ablation in oocytes, we checked the mRNA expression of *SPAST*, *KATNAL1*, and *FIGNL1*. The mRNA levels of *FIGNL1* were significantly diminished but not those of *SPAST* and *KATNAL1*. A typical weak signal of Fignl1 at the spindle pole was found in PNMC-exposed oocytes. This finding is consistent with that of a previous study, whereby Fignl1 knockdown caused spindle defects characterized by an abnormal length/width ratio and decreased microtubule density [27]. Therefore, we speculate that the defective spindle after PNMC exposure is partially due to the abnormal localization of Fignl1.

Previously published data validated that irregular spindle organization induced by MTSP ablation causes abnormal mitochondrial distribution [28]. As a ubiquitously dynamic organelle, the movement of mitochondria is dependent on the microtubules during the cell cycle [29]. Disassembly of the microtubules totally disrupts mitochondrial fusion and fission, and bioenergetics [30]. As PNMC exposure induced the aberrant organization of spindle and mis-localization of MTSPs, we investigated the mitochondrial function in control and PNMC-exposed groups. PNMC treatment led to abnormal mitochondrial distribution. The spindle defects likely disrupt mitochondrial fusion and fission, thereby affecting mitochondrial distribution [31]. Accordingly, the significantly diminished mRNA levels of the fission genes, *FIS1* and *DRP1*, and fusion genes, *MFN1* and *OPA1*, confirm this hypothesis. MMP, another important indicator of mitochondrial function, also decreases sharply in PNMC-exposed oocytes. As MMP drives ATP synthesis, we observed decreased ATP levels following PNMC exposure in coordination with the decline in MMP. Mitochondria provide ATP through the coupling of the electron transport chain to OXPHOS, including enzyme Complex I to V [32]. The mRNA levels of *SDHA* (Complex II), *UQCRC2* (Complex III), and *ATP5A1* (Complex V) were significantly decreased. PNMC exposure caused severely abnormal mitochondrial function, which may be attributed to the defective spindle architecture.

Oocytes extensively depend on optimal mitochondrial functions for their maturation [33]. In mammals, there are more than 100,000 mitochondria per healthy oocyte but in somatic cells, mitochondrial numbers range from a few hundred to thousands [34]. Mitochondria undergo dynamic redistribution during meiotic maturation and are concentrated around the spindle during the metaphase of the first meiosis [35]. Mitochondrial distribution is intimately associated with the meiotic spindle, and scarce ATP production results in abnormalities in the meiotic spindle, indicating the necessity of ATP for spindle organization [36]. Experimental data have directly confirmed that the ablation of mitochondrial-associated genes seriously damages spindle architecture and meiosis progression [37,38]. Therefore, the close crosstalk between the spindle and mitochondria is vital for the maturation and development of oocytes. Furthermore, mitochondria have long been considered sensitive targets of several environmental toxicants, such as pesticides, plasticizers and fungicide [39,40]. Upon uptake into a eukaryotic cell, most toxicants are oxidized, leading to serious ROS accumulation and oxidative stress [41]. Moreover, the mtDNA is devoid of histones, so it is more sensitive to oxidative damage than nuclear DNA [42]. When stress response mechanisms are overloaded by toxicant exposure, cells trigger mitochondria-mediated apoptosis [43]. PNMC exposure can induce apoptosis in various cells and tissues [44]. These findings are consistent with our results. The mRNA levels of the pro-apoptotic genes, *BAX* and *CASPASE3*, were significantly up-regulated; however, that of the anti-apoptotic gene, *BCL-2*, was remarkably down-regulated in the PNMC-exposed oocytes. Annexin-V staining directly confirmed that the number of apoptotic oocytes was significantly elevated in the PNMC-exposed group. We inferred that PNMC exposure induced oocyte apoptosis, likely due to mitochondrial dysfunction.

In brief, PNMC exposure caused defective spindle stability and mitochondrial function. Given the close crosstalk between the spindle apparatus and mitochondria during meiotic maturation, we could not definitively conclude if the abnormal spindle instability resulted in mitochondrial dysfunction, or vice versa [45,46]. However, our findings directly validated that PNMC triggered early apoptosis in oocytes, likely due to mitochondrial dysfunction.

## 4. Materials and Methods

### 4.1. Animal Statement

All experimentation in this study conferred the guidelines set by the Animal Care and Use Committee of Hubei Academy of Agricultural Sciences (HBAAS-2023-014). The 3–4 weeks old female Kunming mice were obtained locally and bred at the experimental center of Hubei Academy of Agricultural Sciences. The animals had access to water and food ad libitum and were reared under a 12 h light/dark cycle at 15–20 °C.

### 4.2. Antibodies and Reagents

Rabbit anti-Fignl1 polyclonal antibody (Cat# NBP2-47456) was procured from Novus Biologicals (Centennial, CO, USA); mouse anti-α-tubulin-FITC antibody (Cat# F2168) was purchased from Sigma Chemical Company (St. Louis, MO, USA); DyLight 549-conjugated goat anti-rabbit IgG (H + L) from Abbkine Biotechnology (San Diego, CA, USA), and IBMX (Cat# HY-12318) was obtained from MedChemExpress Company (Monmouth Junction, NJ, USA).

### 4.3. In Vitro Maturation of Murine Oocyte

Specifically, 3–4 weeks old female Kunming mice were treated with 8 IU of pregnant mare serum gonadotropin and sacrificed by cervical dislocation after 44–48 h of injection. For GV oocyte collection, cumulus-oocyte complexes were isolated from ovarian by mechanically puncturing with an insulin needle, followed by repeated mouth-controlled pipetting to strip off the cumulus cells. GV oocytes were harvested in a M2 medium supplemented with 50 µM IBMX. During in vitro maturation, nearly 30 GV oocytes were grown in a 50 µL M2 drop covered with 2.5 mL mineral oil at 37 °C in an incubator with 5% CO_2_. The oocytes were incubated for 0, 2, 8, and 14 h, corresponding to the GV, GVBD, MI, and MII stages, respectively [47].

### 4.4. Drug Treatment

After dissolving in dimethyl sulfoxide (DMSO), the PNMC (Cat# 2042-14-0, Sigma) stock solution (1 mM) was diluted in the M2 medium to obtain working concentrations, adhering to a DMSO concentration of <0.1%. For the nocodazole (Cat# 31430-18-9, Sigma) treatment, the nocodazole stock solution prepared in DMSO (6 mg/mL, Sigma) was diluted in the M2 medium to obtain a working concentration of 6 µg/mL. For treatment, MI oocytes were incubated with 6 μg/mL nocodazole for 10 min to deploy the spindle apparatus.

### 4.5. RNA Isolation and Real-Time PCR

Total RNA was obtained from 50 oocytes using the RNAqueous Microkit (AM1931, Thermo Fisher Scientific, Waltham, MA, USA) and DNase I (18047019, Thermo Fisher Scientific) was used to inhibit contamination from genomic DNA. Reverse transcription was conducted using the SuperScript IV kit (12594100, Thermo Fisher Scientific). The RNA levels of target genes were quantified using the SsoFast EvaGreen Supermix (172-5200, Bio-Rad, Hercules, CA, USA). Real-time PCR was performed on QuantStudio 3 system (Thermo Fisher Scientific). The 2^−ΔΔCT^ method was used to calculate relative expression. The primers used for Real-time PCR are listed in Table 1.

### 4.6. Immunofluorescent Staining

GV oocytes grown in the M2 medium for 8 h (MI stage) were fixed with 4% paraformaldehyde and permeabilized with 0.5% Triton X-100 in PBS for 50 min. Oocytes were blocked with 1% BSA in the washing solution (0.1% Tween-20 and 0.01% Triton X-100 in PBS) at 15–20 °C for 1 h. Subsequently, the oocytes were directly incubated overnight at 4 °C with the Fignl1 primary antibody (1:100) or with α-tubulin-FITC antibody (1:200) for 1 h at 37 °C. After thorough washing, oocytes were incubated at 37 °C for 1 h with the corresponding secondary antibody. Subsequently, oocytes were stained with DAPI (1 µg/mL) at 15–20 °C for 5 min to visualize nuclear DNA. The images were captured using a confocal laser microscope (Zeiss LSM 810 META, Carl Zeiss Imaging, Jena, Germany).

### 4.7. Detection of ROS in Oocytes

The levels of intracellular ROS in oocytes were examined using the ROS assay kit (S0033S, Beyotime, Shanghai, China). Control and PNMC-exposed oocytes were grown for 8 h at 37 °C in an incubator with 5% CO_2_. PNMC was removed by thorough washing using the M2 media. Next, oocytes were transferred to the M2 media supplemented with 5 μM DCFH-DA and incubated for 30 min. Oocytes were washed three times in PBS-PVA media and placed on confocal coverglass-bottom Petri dishes for immediate observation. All oocytes were scanned using the same parameters by confocal microscopy. The fluorescence intensity was quantified using the LSM Image Browser software (ZEN 2011).

### 4.8. Determination of ATP Levels

The relative concentrations of ATP were measured using an ATP assay kit (S0026, Beyotime, Shanghai, China) following the manufacturer’s instructions. Briefly, control and PNMC-exposed oocytes were grown for 8 h at 37 °C in an incubator with 5% CO_2_. Subsequently, 10 oocytes were lysed in 4 µL lysis buffer by rapid freeze–thaw cycles in liquid nitrogen twice, and 16 µL of the ATP checking solution (1:4 ratio with the lysis buffer) was added. The volumes of lysis buffer and checking solution were calculated depending on the number of oocytes. After 3–5 min of incubation at 15–20 °C, samples were immediately transferred into a 96-well plate in the dark and assessed by an automatic multifunction chemiluminescent analysis system (Varioskan LUX, Thermo Fisher Scientific).

### 4.9. MMP Assay

MMP was determined using a JC-1 kit (Shanghai, C2006, Beyotime, Shanghai, China). Briefly, control and PNMC-exposed oocytes were grown for 8 h at 37 °C in an incubator with 5% CO_2_. PNMC was removed by a thorough washing of the oocytes in the M2 media. Oocytes were incubated with 5 μM JC-1 (diluted in M2) for 30 min at 37 °C. After washing in PBS-PVA thrice, oocytes were scanned by confocal microscopy using the same parameters.

### 4.10. Mitochondrial Distribution

Mito-Tracker Red (C1035, Beyotime, Shanghai, China) was used to determine the mitochondrial distribution in the oocytes. After 8 h of culture in vitro, control and PNMC-exposed MI oocytes were stained with 200 nM Mito-Tracker Red in the M2 medium at 37 °C and 5% CO_2_. After 30 min of incubation, oocytes were washed thrice in PBS-PVA. Subsequently, control and PNMC-exposed oocytes were immediately scanned using the same parameters by confocal microscopy.

### 4.11. Apoptotic Staining

The Annexin V-FITC kit (C1062S, Beyotime, Shanghai, China) was applied to detect the oocytes undergoing early apoptosis. After M2 washes, a mixture of 90 μL binding buffer and 10 μL of Annexin-V-FITC was used to stain the control and PNMC-exposed MI oocyte at 15–20 °C for 20 min. Oocytes were then washed thrice with PBS-PVA and the number of apoptotic oocytes was assessed immediately using a confocal laser scanning microscope.

### 4.12. Statistical Analysis

For the rates of PBE, cell death, oocytes with Annexin V, the significant difference between PNMC treatment and the control were evaluated by Fisher’s exact test using the GraphPad Prism software (version 8.0, San Diego, CA, USA). Data are presented as boxplot of at least three biological experiments. *p*-value < 0.05 was considered statistically significant. For spindle intensity, length, width and area, mRNA level, ATP, JC-1 and ROS, the significant difference between PNMC treatment and the control were examined by independent sample *t*-tests using GraphPad Prism software. Data are presented as the mean ± SEM of at least three biological experiments. *p*-value < 0.05 was considered statistically significant.

## 5. Conclusions

In this study, we investigated the toxic mechanisms of PNMC on mouse oocyte maturation. Our results indicates that a low-dose PNMC exposure could disrupt oocyte meiotic maturation and quality, by disturbing spindle architecture and mitochondrial function, which could finally induce early apoptosis.

## Figures and Tables

**Table 1:** Primer sequences used for real time.

Gene	Gene ID	Primer Sequences (5′ to 3′)	Product (bp)
*ATP5A1*	NM_007505.2	F: AATCTCCATGCCTCTAACACTCGAC R: GCAATACCATCACCAATGCTTAAC	143
*ATP6*	NP_904333.1	F: TCCCAATCGTTGTAGCCATC R: TGTTGGAAAGAATGGAGTCGG	91
*BAX*	NM_007527.3	F: ATGCGTCCACCAAGAAGCTGAG R: CCCCAGTTGAAGTTGCCATCAG	166
*BCL2*	NM_009741.5	F: ATGATAACCGGGAGATCGTG R: GACGGTAGCGACGAGAGAAG	294
*CASPASE3*	NM_001284409.1	F: GACTGGGATGAACCACGACCC R: TCTGACTGGAAAGCCGAAAC	205
*COX1*	YP_001686700.1	F: TTTTCAGGCTTCACCCTAGATGA R: CCTACGAATATGATGGCGAAGTG	62
*CYTB*	YP_220562.1	F: ATTCCTTCATGTCGGACGAG R: ACTGAGAAGCCCCCTCAAAT	228
*DRP1*	NM_152816.4	F: TCCCAATTCCATTATCCTCGC R: CATCAGTACCCGCATCCATG	149
*FIS1*	NM_001347504.1	F:CAAAGAGGAACAGCGGGACT R:ACAGCCCTCGCACATACTTT	95
*GAPDH*	NM_001289726.2	F:TCGGAGTGAACGGATTTGGC R:TGACAAGCTTCCCGTTCTCC	189
*GPX4*	NM_001367995.1	F: AAATCAAGGAGTTTGCAGCCGG R: TTCTCTATCACCTGGGGCTCCT	229
*GPX6*	NM_145451.3	F: GCCCAGAAGTTGTGGGGTTC R: TCCATACTCATAGACGGTGCC	129
*MFN1*	NM_024200.5	F: TATTGGCGAGGTGCTGTCTC R: TCTGCAGTGAACTGGCAATG	71
*ND1*	YP_220550.1	F: TGCACCTACCCTATCACTCA R: GGCTCATCCTGATCATAGAATGG	148
*NDUFV1*	NM_133666.3	F: GCGGGTATCTGTGCGTTTCA R: GCGCCCATACAGGTTGGTAAAG	103
*OPA1*	NM_001199177.2	F:ACCTTGCCAGTTTAGCTCCC R: TTGGGACCTGCAGTGAAGAA	82
*SDHA*	NM_023281.1	F: GCGTATGTGCTGGCTAGCTT R: AAGCCAATCCCTCAGAGACA	121
*SOD1*	NM_011434.2	F: GAGAGCATTCCATCATTGGCCG R: CGCAATCCCAATCACTCCACAG	134
*SOD2*	NM_013671.3	F: CAGACCTGCCTTACGACTATGG R: CTCGGTGGCGTTGAGATTGTT	113
*UQCRC2*	NM_025899.2	F: AACCCGTGGGATTGAAGCAG R: CTGTGGTGACATTGAGCAGGAAC	131

## Data Availability

The data that support the findings of this study are available from the corresponding author upon reasonable request.
